# Structural Analysis of Missense Mutations on the Stability of APOE3 and APOE4

**DOI:** 10.3390/genes16121509

**Published:** 2025-12-16

**Authors:** Malcolm Anthony, Yixin Xie, Jahn N. O’Neil, Shaolei Teng

**Affiliations:** 1 Department of Biology, Howard University, Washington, DC 20059, USA; malcolm.anthony@bison.howard.edu; 2 College of Computer Science and Software Engineering, Kennesaw State University, Marietta, GA 30060, USA; 3 Department of Physiology & Biophysics, Howard University College of Medicine, Washington, DC 20059, USA; jahn.oneil@howard.edu

**Keywords:** APOE, computational saturation mutagenesis, protein stability, molecular dynamics simulation, Alzheimer’s disease

## Abstract

**Background/Objectives:** Apolipoprotein E (APOE) plays a central role in lipid transport and neuronal cholesterol metabolism. Among its three major isoforms (APOE2, APOE3, and APOE4), the APOE4 variant is the strongest genetic risk factor for late-onset Alzheimer’s disease (AD). However, the structural consequences of specific APOE mutations on protein stability remain poorly understood. **Methods:** Here, we performed computational saturation mutagenesis and molecular dynamics simulations on the non-lipidated N-terminal fragments of APOE3 and APOE4 to examine how missense mutations affect their structural stability. **Results:** Based on the folding energy (ΔΔG) calculations, mutations G165W and L155W were particularly destabilizing in APOE4. Molecular dynamics analyses showed that these mutations altered local flexibility and compactness, particularly within the helix 4 region, a key structural element for maintaining APOE’s structural integrity. **Conclusions:** Our findings, which are state dependent and hypothesis generating, highlight isoform-dependent differences in protein stability and identify regions of structural vulnerability within APOE. These insights enhance our understanding of APOE’s conformational dynamics and may inform future studies on its role in neurodegenerative disease mechanisms.

## 1. Introduction

Alzheimer’s disease (AD) is the most common neurodegenerative disorder. It currently affects over 7 million Americans, and that number is expected to increase with population aging [1]. The disease is characterized by progressive cognitive decline and is pathologically marked by amyloid-β plaque accumulation, neurofibrillary tau tangles, synaptic dysfunction, and neuroinflammation [2]. Despite decades of research, effective therapeutic options remain limited, and understanding the genetic and molecular contributors to disease progression is essential.

Apolipoprotein E (APOE) is a lipid transport protein encoded by the *APOE* gene on chromosome 19, which plays a central role in cholesterol and lipid metabolism. In the central nervous system, APOE is crucial for lipid trafficking between neurons and glial cells [3]. In addition to the three common human isoforms (APOE2, APOE3, and APOE4), three rare electrophoretic isoforms—APOE1, APOE5, and APOE7—have also been reported [4]. Of the three common human APOE isoforms—APOE2, APOE3, and APOE4—APOE4 is the strongest genetic risk factor for late-onset AD [5], while APOE2 appears protective, and APOE3 is considered neutral [6]. These isoforms differ only at positions 112 and 158, but even these small changes profoundly impact structure, receptor binding, and downstream cellular responses [7].

Importantly, specific APOE mutations beyond the isoform-defining residues have been shown to influence protein stability and function [8,9]. Alterations in APOE structure can affect lipid binding, receptor interactions, and aggregation propensity, factors that contribute to amyloid-β accumulation and neurotoxicity. However, many APOE variants remain poorly characterized, and the structural consequences of potentially stabilizing or destabilizing mutations, especially within the context of APOE4, are not well understood.

One emerging area of interest is the interaction between APOE and TREM2, a microglial receptor involved in phagocytosis and immune responses in the brain [10,11,12]. TREM2 variants, such as the rs75932628 (encoding R47H) variant, are also associated with increased AD risk [13], and studies suggest that APOE-TREM2 binding plays a critical role in modulating microglial activation and amyloid clearance [11,12]. Disruptions to this interaction may therefore contribute directly to AD pathogenesis, especially in APOE4 carriers.

While prior studies have examined APOE isoforms and their impact on AD risk [14], few have systematically explored the effects of specific point mutations on APOE structure and its ability to interact with TREM2. Recent computational studies have begun to address the APOE dynamics in discoidal lipoproteins and lipid nanodiscs [15,16,17], but these studies focus on lipidated APOE or model lipid nanodisc systems rather than systematic mutational scanning of APOE crystal structures and isoform-specific effects. Despite these advances, there remains limited information about the dynamic behavior of APOE variants under physiological conditions [18,19].

In this study, we combine saturation mutagenesis, stability prediction tools, and molecular dynamics simulations on the non-lipidated N-terminal fragments of APOE3 and APOE4 to identify and characterize structurally impactful mutations in both isoforms. We focus in particular on the APOE4 isoform due to its pathogenic relevance and evaluate how specific variants affect protein stability and dynamics. Our findings reveal distinct stabilizing and destabilizing mutations, such as G165W and L155W, that may influence APOE-TREM2 interactions and provide new insights into the structural underpinnings of AD risk.

## 2. Materials and Methods

### 2.1. Sequence and Structural Alignment

The amino acid sequences of APOE3 and APOE4 were aligned using Clustal Omega (version 1.2.4) to highlight key residue differences between the N-terminal fragments of the two isoforms. Corresponding three-dimensional structures were obtained from the Protein Data Bank (PDB IDs: 1NFN [20] for APOE3 and 1GS9 [21] for APOE4). The 1GS9 PDB structure was never published, which explains its different formatting compared with 1NFN in the References section. These APOE structures are both lipid-free and N-terminal fragments of the mature APOE protein. These APOE3 and APOE4 structures were used for further computational analyses based on the current selection of APOE PDB structures and factors such as residue coverage, mutations already introduced, and complex formation. Structural alignment was performed using PyMOL, and the root-mean-square deviation (RMSD) between the aligned structures was calculated to quantify conformational differences. Residue numbering refers to the mature APOE sequence; to compare with clinical reports using pre-APOE numbering (which include the 18 aa signal peptide), subtract 18 residues (e.g., clinical L173P = mature L155P).

### 2.2. Computational Saturated Mutagenesis

We performed in silico saturated mutagenesis on the whole non-lipidated APOE3 (1NFN) and APOE4 (1GS9) X-ray crystal structure N-terminal fragments, which were acquired from the Protein Data Bank, along with their corresponding FASTA sequences. The full-length APOE3 (2L7B) structure was mutated as well for the purpose of comparison, since 1NFN and 1GS9 only possess N-terminal fragments of the APOE variants [22]. Saturated mutagenesis was applied to model 1 of the 2L7B NMR ensemble (chain A). A custom Python script was utilized, in conjunction with FoldX [23], to generate all possible mutations within the experimental structures of APOE3 (1NFN) and APOE4 (1GS9). The script is available in the Supplementary Information (Supplementary File S1). Every amino acid residue of the structures was mutated into all 19 of the other amino acid residues, resulting in the generation of 2508 missense mutations for APOE3 (1NFN), 2736 missense mutations for APOE4 (1GS9), and 5681 missense mutations for full-length APOE3 (2L7B).

### 2.3. Energy Calculation

The energy calculations were conducted using FoldX. The effects of the mutations on the stability of the proteins were estimated by the folding energy change (ΔΔG), which is calculated using this formula: ΔΔG = ΔG_mut − ΔG_wt. ΔG is the Gibbs free energy difference between the folded and unfolded states of one protein variant. ΔΔG is the difference between two ΔG fold values, one for the mutant and one for the wild type. WT refers to the wild-type form, the commonly occurring version of a protein. MUT denotes the mutant protein carrying the amino acid substitution(s). The protein structures were repaired using the RepairPDB command, and the BuildModel command was used for stability analysis. A negative ΔΔG value indicates that the mutation may stabilize the protein, and a positive value indicates that it may destabilize the protein.

### 2.4. Solvent-Accessible Surface Area (SASA) Analysis

Per-residue SASA was computed for APOE residues 130–165 on 1NFN, 1GS9, and 2L7B using PyMOL (v3.0.3) get_area with a 1.4 Å probe and dot_solvent = 1 [24]. Prior to calculation, structures were prepared by removing waters/ions/ligands and applying FoldX RepairPDB. Atomic SASA values were summed per residue to obtain per-residue SASA (Å^2^) in PDB numbering. Bar plots used identical y-axes across structures to enable direct comparison.

### 2.5. Supplementary Stability Predictions via DynaMut2 and DUET

We submitted the most destabilizing and stabilizing APOE mutations identified in our analysis of 1NFN and 1GS9, along with the common APOE isoform-defining missense variants (allele frequency > 1%) from the gnomAD database, to the DynaMut2 [25] and DUET [26] servers to compare their predicted ΔΔG values with those obtained from FoldX. The calculation of the ΔΔG values via DynaMut2 is carried out by integrating graph-based signatures of the WT residue environment with Normal Mode Analysis-derived dynamics (ENCoM/Bio3D parameters). The calculation of ΔΔG values via DUET is carried out by combining mCSM (graph-based 3D environment signatures) and SDM (environment-specific substitution propensities) in a consensus model. Two variants were identified in gnomAD: Cys112Arg (rs429358) and Arg158Cys (rs7412). This allowed us to assess how the known isoform-defining mutations compare to the mutations under study across all 3 APOE structures (1NFN, 1GS9, and 2L7B).

### 2.6. Molecular Dynamics Simulation

We performed molecular dynamics simulations of the WT APOE3, APOE4, and mutant APOE N-terminal fragments in explicit solvent using GROMACS 2023 [27], a widely used open-source package for biomolecular simulations, on Ubuntu 24.04. Each protein was placed in a triclinic box and solvated with TIP3P water molecules, with a minimum of 10 Å padding around the solute to ensure full hydration. The system was then neutralized and supplemented with approximately 150 mM NaCl to reflect physiological ionic strength.

Simulations were conducted in the following sequence: energy minimization using the steepest descent algorithm until convergence (maximum force < 1000 kJ/mol/nm), NVT equilibration for 100 ps at 310 K using the V-rescale thermostat, NPT equilibration for 100 ps at 1 atm using the Parrinello–Rahman barostat, followed by production MD simulations with a 2 fs time step under periodic boundary conditions.

To assess structural dynamics, we performed three independent production runs of the APOE4 system (PDB ID: 1GS9) to evaluate reproducibility and statistical stability. For other systems, including APOE3 (PDB ID: 1NFN) and all mutant variants, a single production run was conducted due to time and resource constraints. While multiple replicates would provide greater statistical confidence, all runs were visually inspected for equilibration, and standard analyses were conducted to ensure structural and energetic consistency across trajectories.

Representative structures at selected time points (starting structure, maximum RMSD, and increased β-sheet content) were extracted from the trajectories and visualized in PyMOL.

## 3. Results

### 3.1. Sequence and Structural Alignment of APOE3 and APOE4

**Figure 1:**
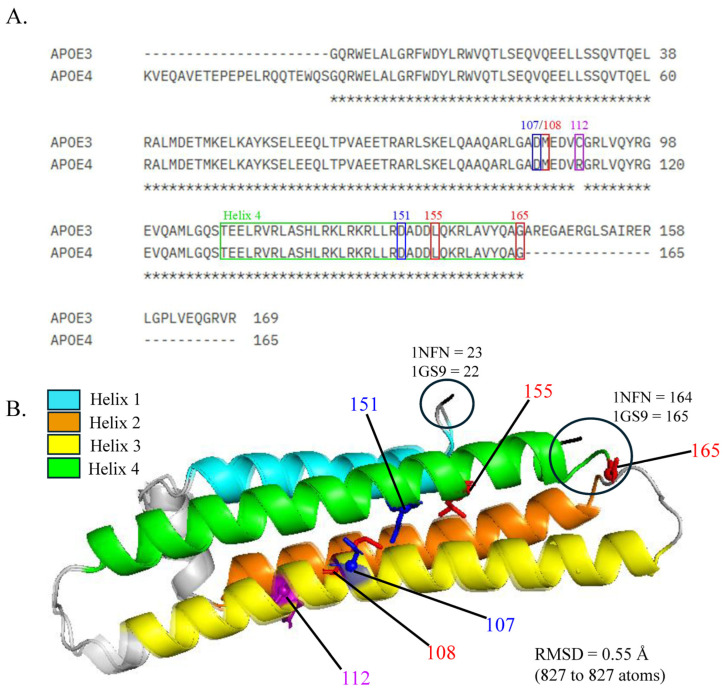
Sequence and structural alignment of APOE3 and APOE4. (**A**) The alignment of the APOE3 (1NFN) and APOE4 (1GS9) structures, with the helix 4 region (green), position 112 (purple), and the top 2 stabilizing (blue) and destabilizing (red) mutations highlighted. (**B**) The structural alignment of APOE3 and APOE4 resulted in an RMSD of 0.55 Å. Helix 1 (residues 24–42), helix 2 (residues 54–81), helix 3 (residues 87–122), helix 4 (residues 130–164), position 112 (purple), and the top 2 stabilizing (blue) and destabilizing (red) mutations are highlighted. The N- and C-terminal residues are denoted by the black spheres and labeled with their residue numbers.

The key isoform differences and the top mutations identified through saturated mutagenesis (those with the greatest mean ΔΔG and alanine values) are identified in the aligned sequences and structures of APOE3 and APOE4 (Figure 1) to emphasize their locations relative to the helix 4 region of APOE, which has been implicated in TREM2 binding. Notably, mutations at positions 151 and 155 reside within helix 4, while those at positions 107 and 108 lie outside this region. Mutation 165 is located just beyond helix 4, and position 112 also falls outside this region. Structural alignment of APOE3 and APOE4 using PyMOL resulted in a root-mean-square deviation (RMSD) of 0.55 Å, with 827 atoms perfectly matched between the two isoforms.

### 3.2. Computational Analysis of the Effect of Missense Mutations on APOE3 and APOE4

**Figure 2:**
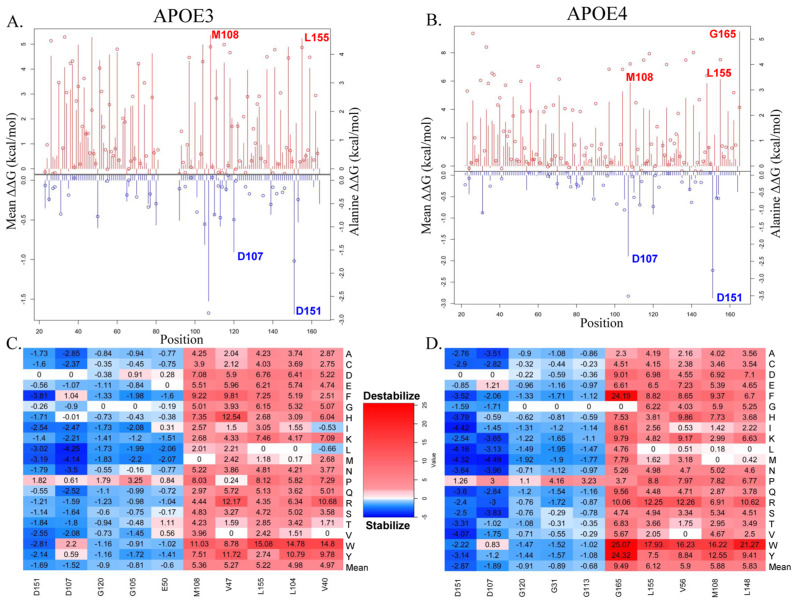
Saturated mutagenesis results. (**A**,**B**) The line charts of APOE3 and APOE4, respectively, with the lines representing the mean ΔΔG values (kcal/mol) and the spheres representing the alanine values of each residue. (**C**,**D**) The heat maps correspond to the line charts of APOE3 and APOE4, respectively. The top destabilizing and stabilizing residues of each protein were chosen based on their mean ΔΔG (kcal/mol) and alanine values, with red representing destabilizing missense mutations and blue representing stabilizing missense mutations.

The saturated mutagenesis of APOE3 and APOE4 revealed the top destabilizing and stabilizing missense mutations of both proteins. It resulted in 2508 missense mutations for APOE3 and 2736 missense mutations for APOE4. Out of the 2508 missense mutations for APOE3, 1710 (68.18%) were destabilizing, and 798 (31.82%) were stabilizing (Supplementary Table S1). Out of the 2736 missense mutations for APOE4, 1924 (70.32%) were destabilizing, and 812 (29.68%) were stabilizing (Supplementary Table S2). The mutation sites shown at the bottom of Figure 2B,C were selected because they exhibited the highest mean ΔΔG values (kcal/mol) among all the sites analyzed. We chose to highlight mutations M108W and L155W in both APOE3 and APOE4, D107L and D151F in APOE3, and D107M and D151I in APOE4 in Figure 1A,B because these were among the top mutation sites (based on their mean ΔΔG values) identified in the APOE3 (1NFN) and APOE4 (1GS9) structures, with the exception of G165W, which only appeared in APOE4. This mutation lies just outside of the helix 4 region of APOE, a region important for TREM2 interaction, and boasted an incredibly high change in Gibbs free energy (ΔΔG) of 25.07 kcal/mol. This indicates a possible increase in the instability of APOE4. The L155W mutation, positioned within helix 4 of APOE, exhibited a ΔΔG of 17.93 kcal/mol and 15.08 kcal/mol in APOE4 and APOE3, respectively. Both changes in Gibbs free energy indicate destabilizations of the protein as well, along with the M108W mutation in APOE4 and M108W mutation in APOE3, with ΔΔG values of 16.22 kcal/mol and 11.03 kcal/mol, respectively. The top two stabilizing mutations of APOE4 include D151I and D107M, with ΔΔG values of −4.42 kcal/mol and −4.49 kcal/mol, respectively. The top two stabilizing mutations of APOE3 include D151F and D107L, with ΔΔG values of −3.81 kcal/mol and −4.25 kcal/mol, respectively. Position 151 also lies within the helix 4 region of APOE as well, as this region encompasses residues 130–164 [7,28,29]. The other top mutation sites—G120 in both APOE3 and APOE4; G105, E50, V47, L104, and V40 in APOE3; and G31, G113, V56, and L148 in APOE4—shown in Figure 2C,D were not evaluated further, as we chose to focus on the most strongly destabilizing/stabilizing sites that appeared in both structures and to keep the analysis concise.

**Figure 3:**
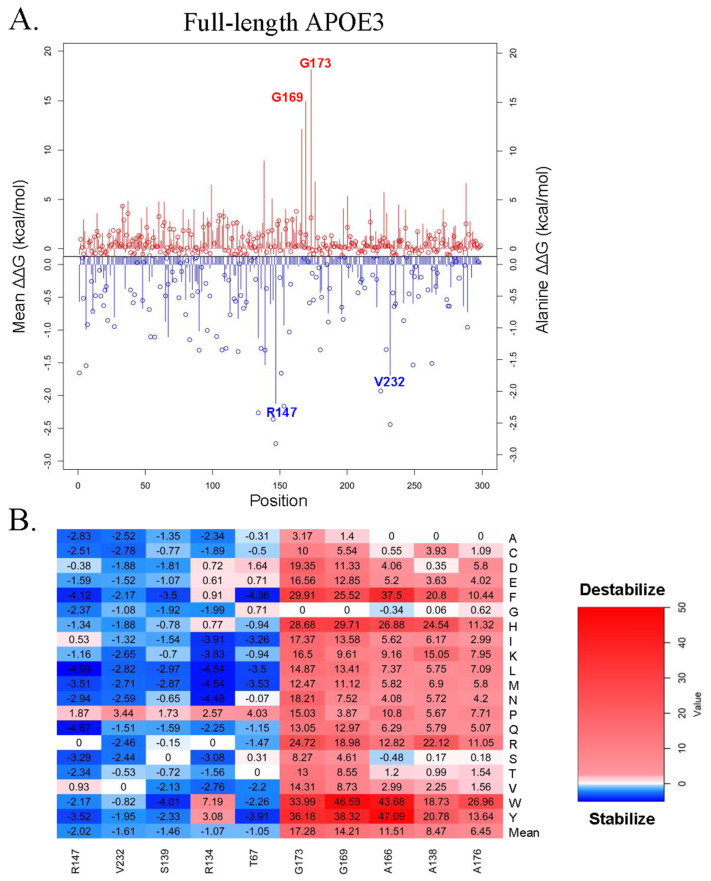
Saturated mutagenesis results. (**A**) The line chart of full-length APOE3 (2L7B), with the lines representing the mean ΔΔG values (kcal/mol) and the spheres representing the Alanine values of each residue. (**B**) The heat map corresponds to the line chart of full-length APOE3 (2L7B). The top destabilizing and stabilizing residues of each protein were chosen based on their mean ΔΔG (kcal/mol) and alanine values, with red representing destabilizing missense mutations and blue representing stabilizing missense mutations.

The saturated mutagenesis of full-length APOE3 (2L7B) yielded 5681 missense mutations. Out of the 5681 missense mutations, 3723 (65.53%) were destabilizing, and 1958 (34.47%) were stabilizing (Supplementary Table S3), comparable to the percentages observed for APOE3 (1NFN) and APOE4 (1GS9). Similar to Figure 2C,D, the mutation sites shown at the bottom of Figure 3B were selected because they exhibited the highest mean ΔΔG values (kcal/mol) among all the sites analyzed. We chose to highlight mutations G173, G169, R147, and V232 in Figure 3A because these were among the top mutation sites (based on their mean ΔΔG values) identified in the full-length APOE3 (2L7B) structure. While the exact top residues differed from the N-terminal models, the enriched regions were similar. Several of the most destabilizing 2L7B sites (G173, G169, and A166) cluster near the helix-4/hinge boundary and are adjacent to the APOE4 hotspot G165 in 1GS9. L155 (highlighted in both N-terminal structures) falls within the same neighborhood. Conversely, A138 is within the canonical LDL-receptor binding region (~135–150), aligning with L148 in APOE4. The top 2L7B destabilizing hits localize to the N-terminal domain or hinge region (e.g., G173, G169, A166, and A176), consistent with our N-terminal analyses. Note that 2L7B is an engineered non-lipidated model containing five C-terminal stabilizing mutations, which could dampen apparent CTD sensitivity and bias extremes toward the NTD/hinge region. Among stabilizing predictions, all but one (V232) occur in the NTD (e.g., R147, S139, and R134). These residues lie near stabilizing features seen in 1NFN/1GS9 (e.g., D151 and G120), and several (S139 and R147) fall within the LDLR-binding epitope window. In 2L7B, aromatic substitutions at hinge-proximal Gly/Ala residues exhibited the largest predicted destabilizations, which include A166Y (47.09 kcal/mol), G169W (46.59 kcal/mol), A166W (43.68 kcal/mol), G169Y (38.32 kcal/mol), G173Y (36.18 kcal/mol), and G173W (33.99 kcal/mol). These values reflect model-based predictions rather than experimental free energies and should be interpreted as relative effect sizes.

**Figure 4:**
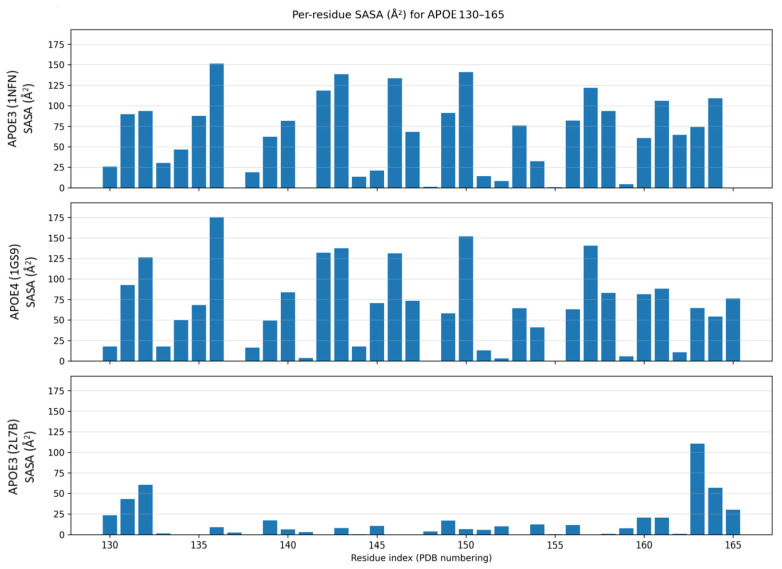
Helix 4 exposure across structures. Per-residue SASA (Å^2^) for APOE residues 130–165 in 1NFN, 1GS9, and 2L7B (engineered full-length, non-lipidated model). Higher SASA indicates greater solvent exposure; identical *y*-axes enable direct comparison. Exposure is broadly reduced in 2L7B across residues ~136–160, with exposure largely retained at the termini, underscoring state dependence.

Per-residue SASA in Figure 4 showed high exposure across residues 130–165 in the N-terminal models of 1NFN and 1GS9 but substantial burial of residues ~136–160 in full-length 2L7B, with exposure mainly retained at residues 130–133 and residues 163–165. These patterns indicate that helix 4 accessibility—and thus any receptor-facing epitope in this window—is strongly state dependent.

The apparent differences in ΔΔG values between FoldX, DynaMut2, and DUET stem from the inclusion of additional factors—such as protein dynamics and flexibility—by DynaMut2 and DUET (see Table 1). Despite these variations in numerical output, the overall classification of mutations as stabilizing or destabilizing was largely consistent across the tools. For both APOE3 and APOE4, the top two most destabilizing mutations identified by FoldX (and G165W in APOE4) were also labeled as destabilizing by DynaMut2 and DUET, except for the M108W mutation in APOE3, which was labeled as stabilizing via DynaMut2. Similarly, the D151I, D107M, and D107L mutations were consistently labeled as stabilizing across all three tools.

An exception was the D151F mutation, which was predicted as stabilizing by FoldX but classified as destabilizing by DynaMut2 and DUET, likely due to the consideration of dynamic structural changes not captured by FoldX.

Predicted effects for full-length APOE3 (2L7B) were largely consistent with APOE3 (1NFN) and APOE4 (1GS9) across FoldX, DynaMut2, and DUET. Previously noted hotspots (e.g., G165W in APOE4/2L7B and L155W in all three structures) were uniformly predicted destabilizing by all tools. M108W was destabilizing across all tools for 2L7B, mirroring APOE4, and D151I was stabilizing across all tools for APOE4 and 2L7B. Notable differences included D151F, which was destabilizing across all tools for 2L7B (vs. mixed predictions in 1NFN), and D107M/D107L, which were stabilizing across all tools in the NTD models but shifted to destabilizing for 2L7B in most tools (with FoldX still predicting D107L as stabilizing). These differences likely reflect full-length packing/NTD-CTD context in 2L7B rather than tool-specific effects. We included the common APOE isoform-defining missense variants from gnomAD: Cys112Arg (rs429358) and Arg158Cys (rs7412), which together define the APOE2, APOE3, and APOE4 isoforms. These are the common population variants (>1% allele frequency) with well-documented effects on Alzheimer’s disease risk (APOE2 generally protective, APOE3 neutral, and APOE4 risk increasing). For Cys112Arg, FoldX predicts slight stabilization (ΔΔG = −0.53 kcal/mol), whereas DynaMut2 (−0.98 kcal/mol) and DUET (−0.912 kcal/mol) predict moderate destabilization. For Arg158Cys, all three tools indicate destabilization (FoldX = 1.05 kcal/mol; DynaMut2 = −1.23 kcal/mol; DUET = −1.614 kcal/mol).

### 3.3. Molecular Dynamics Simulations of APOE3 and APOE4 Missense Variants

**Figure 5:**
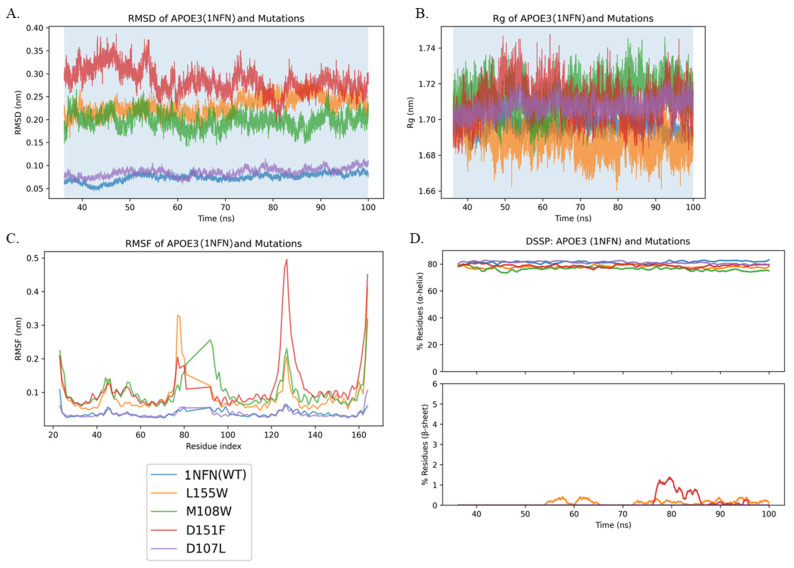
APOE3 (1NFN) and mutants: RMSD, Rg, RMSF, and DSSP. (**A**) RMSD vs. time. (**B**) Radius of gyration (Rg) vs. time. (**C**) RMSF per residue. (**D**) DSSP secondary structure: helix (top) and β-sheet (bottom) vs. time. All traces are from the production window 36.2–100 ns, which excludes early transients and captures post-equilibration behavior. Per-frame DSSP strings were parsed, with helix defined as H + G + I (DSSP labels H: α-helix; G: 3_10_-helix; I: π-helix) and β-sheet defined as E + B (DSSP labels E: extended β-strand; B: isolated β-bridge). Values were converted to percent per frame using the number of residues present and then smoothed with a 1.0 ns moving average. Helix traces overlap at ~78–82% across variants. β -sheet content is ~0% for WT, M108W, and D107L, with brief transients up to ~1–1.5% for L155W and D151F. RMSD/RMSF indicates that D151F is the most perturbed, while L155W trends slightly more compact by Rg. (Panels share consistent coloring across metrics.).

In Figure 5, within the 36.2–100 ns production window, D151F exhibits the largest structural deviation (RMSD ≈ 0.25–0.33 Å) and pronounced RMSF hotspots, while 1NFN and D107L appeared the most stable by RMSD. Radius of gyration (Rg) differences are minimal but consistent: L155W trends are more compact; the other mutants sit close to 1NFN. DSSP shows overlapping helix fractions (~78–82%) for all variants. β-sheet is essentially absent in most runs. Only L155W and D151F exhibit brief, low amplitude β transients (≤~1.5%), while 1NFN (WT), M108W, and D107L are ~0% throughout. Therefore, the four-helix bundle of APOE3 remains stable across variants, with only sporadic, very low β in L155W and D151F.

**Figure 6:**
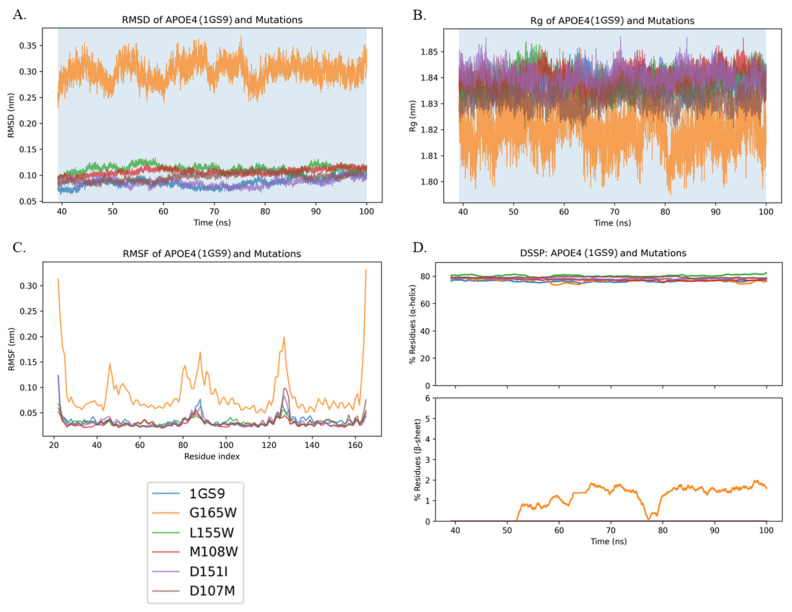
APOE4 (1GS9) and mutants: RMSD, Rg, RMSF, and DSSP. (**A**) RMSD vs. time. (**B**) Radius of gyration (Rg) vs. time. (**C**) RMSF per residue. (**D**) DSSP secondary structure: helix (top) and β-sheet (bottom) vs. time. All traces are from the production window 39.2–100 ns, which excludes early transients and captures post-equilibration behavior. Per-frame DSSP strings were parsed, with helix defined as H + G + I (DSSP labels H: α-helix; G: 3_10_-helix; I: π-helix) and β-sheet defined as E + B (DSSP labels E: extended β-strand; B: isolated β-bridge). Values were converted to percent per frame using the number of residues present then smoothed with a 1.0 ns moving average. Helix curves largely overlap at ~78–82%. β-sheet is near 0% for 1GS9 and all mutants except G165W, which shows transient β up to ~2–3%. RMSD and RMSF identify G165W as the most perturbed (largest RMSD, localized flexibility peaks), while Rg differences are small but show G165W trending slightly more compact. (Panels share consistent coloring across metrics.).

In Figure 6, across the production window (39.2–100 ns), G165W is consistently the most perturbed variant. Its RMSD is noticeably higher and noisier (≈0.25–0.35 Å) than 1GS9 and the other mutants (≈0.07–0.12 Å). The differences in the radius of gyration (Rg) are modest in absolute terms, but G165W trends are slightly more compact (lower Rg), while the rest of the mutations cluster near 1GS9. RMSF exhibits elevated, localized mobility for G165W (termini and mid-chain peaks), whereas the other mutants mostly track 1GS9 with minor bumps. DSSP reveals that α-helix content is highly conserved among all variants at ~78–82%. β-sheet is ~0% for 1GS9 and all mutants except G165W, which exhibits transient β structure up to ~2–3%. Thus, the principal secondary structure difference in APOE4 is occasional low-fraction β formation in G165W, while the other variants retain an essentially pure helical bundle.

**Figure 7:**
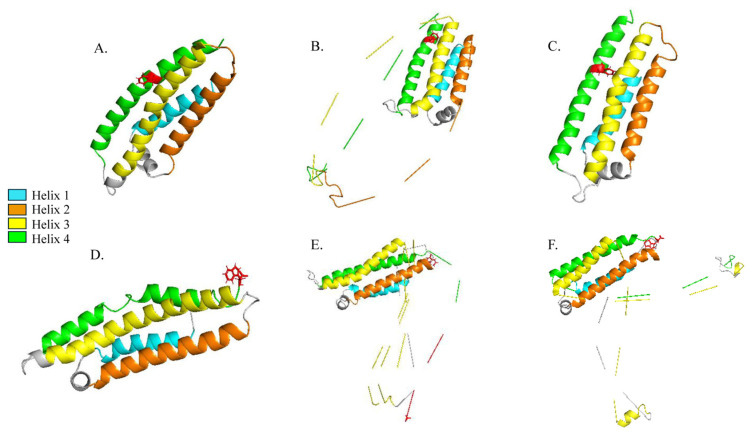
Representative MD snapshots of APOE mutants D151F (top) and G165W (bottom). (**A**–**C**) APOE3 D151F snapshots at (**A**) the starting structure, (**B**) the time point of maximum RMSD (47.1 ns), and (**C**) a frame with detectable β-sheet content (~76.6 ns). (**D**–**F**) APOE4 G165W snapshots at (**D**) the starting structure, (**E**) the time point of maximum RMSD (75.3 ns), and (**F**) a frame with β-sheet content identified by DSSP. Helices 1–4 are colored as in Figure 1, and the mutated residues (D151F and G165W) are shown as red sticks.

Representative MD snapshots for APOE3 mutation D151F and APOE4 mutation G165W at the starting structure, the time point of maximum RMSD, and a time point with increased β-sheet content (based on DSSP) are shown in Figure 7. We chose to extract structural snapshots of D151F and G165W to keep the figure concise and because they exhibited the largest RMSD deviations among their respective groups of simulated variants.

## 4. Discussion

Structural analysis determined that the helix 4 region of APOE (~130–164, mature numbering; add +18 for pre-APOE numbering) contributes to its LDLR-binding region [7,28,29] and has been implicated in TREM2 recognition [11,12], which is an important microglial interaction implicated in AD pathogenesis. APOE-TREM2 pathways include a microglial immune-signaling axis, where APOE serves as a ligand and TREM2 (with DAP12) shapes activation state and cytokine responses. They also include a phagocytosis/clearance route in which APOE-TREM2 binding promotes uptake of apoptotic neurons and debris [11]. Under the lipid-free, N-terminal domain context examined here, L155W or G165W are predicted to affect local dynamics, which could, in principle, change presentation of the candidate TREM2-facing surface. Our DSSP analysis from per-frame strings (helix = H + G + I; β = E +B) shows that α-helix content is tightly conserved, while β-sheet formation is rare and variant specific, with potential implications for how this segment is presented to TREM2. Consistent with this, residues ~136–160 are largely buried in full-length 2L7B, whereas they are highly exposed in the N-terminal models 1NFN/1GS9 (Figure 4). This contrast indicates that helix 4 accessibility—and any putative TREM2-facing epitope within 136–160—is strongly state dependent. However, further experimental validation in lipidated, full-length particles would be needed.

Across both APOE3 (1NFN) and APOE4 (1GS9), secondary structure analysis shows that α-helix content is largely conserved over our MD production windows (APOE4: 39.2–100 ns; APOE3: 36.2–100 ns). In APOE4, G165W is the most perturbed overall (higher RMSD with localized peaks and a slightly more compact Rg) and is the only variant that shows transient β-structure (typically ≤2–3%). 1GS9 (WT), L155W, M108W, and D107M remain ~0% β throughout. In APOE3, helix remains at ~78–82% for all variants; β-sheet is ~0% for 1NFN (WT), M108W, and D107L, while L155W and D151F show only brief, low-amplitude transients (≤1–1.5%). D151F still exhibits the largest RMSD/RMSF deviations, with small differences in Rg. Notably, FoldX predicted D151F as stabilizing, whereas DUET and DynaMut2 labeled it destabilizing, consistent with its larger RMSD (Figure 5A). In 2L7B, all three predictors label D151F destabilizing (Table 1), which is concordant with the elevated RMSD/RMSF in Figure 5A. While RMSD is not a direct readout of thermodynamic stability, the agreement suggests greater conformational perturbation for this variant in the full-length context.

These isoform-specific responses suggest that context matters. One plausible interpretation is that the Arg112-mediated domain interaction in APOE4 subtly repositions the N-terminal four-helix bundle [29], such that M108W is better accommodated in APOE4 than in APOE3 (consistent with the lower RMSD of M108W in APOE4). We view this as a hypothesis rather than a conclusion; additional replicas, longer trajectories, and experiments will be required to test it. Despite the distance of these mutations from the isoform-defining residue at position 112, our results suggest that the Arg112 (APOE4) versus Cys112 (APOE3) background may alter the packing and dynamic coupling of the N-terminal four-helix bundle. In this context, substitutions in helix 4 (e.g., L155W and D151F) and immediately adjacent to it (G165W in APOE4) are likely to experience distinct local environments in APOE3 versus APOE4, leading to isoform-specific stability changes despite their spatial separation from residue 112. Due to the implication of helix 4 (~130–164) in TREM2 recognition [11,12], even moderate changes in local dynamics or occasional β-structure in G165W (APOE4) and very low, brief β transients in L155W/D151F (APOE3) could alter how APOE presents this state-dependent interface, with potential consequences for microglial signaling and contributing to AD pathogenesis [7,30]. Within the lipid-free N-terminal models we analyzed, β-sheet fluctuations—with isoform-dependent features—emerge as one of the more prominent signatures of mutation-induced perturbation, despite an overall conserved helical scaffold.

Clinical genetics also points to the functional sensitivity of helix 4. In lipoprotein glomerulopathy, the APOE ‘Chengdu’ variant (p.L173P; mature L155P) introduces a pathogenic helix-breaking proline within the LDLR-binding region [31]. Although this disease context differs from our CNS focus, it highlights that substitutions at helix 4 positions ~155–164 can materially modify APOE behavior in vivo. Our MD results are consistent with this regional sensitivity: helix content is broadly maintained across variants, but β-sheet fractions—and local dynamics—differ most among mutants in the helix 4 region (notably, G165W in APOE4 shows the only measurable β excursions, whereas APOE3 L155W/D151F exhibit only brief, very low transients). We therefore consider helix 4 a structurally responsive segment whose mutations may modulate how APOE presents interaction surfaces, including those implicated in TREM2 recognition, warranting experimental follow-up, partly because the presentation of this interaction surface is state dependent of APOE.

In terms of AD pathogenesis, the results of this study may motivate structure guided strategies that target APOE helix 4 and its presentation to receptors (e.g., TREM2), aiming to stabilize favorable conformations or lessen pathological ones. They also emphasize the value of variant-focused studies linking APOE mutations to altered dynamics (as shown here and elsewhere), which could help identify subgroups most likely to benefit from targeted interventions.

### Limitations

Our analyses rely on non-lipidated N-terminal domain structures of APOE3 and APOE4 (e.g., 1NFN and 1GS9), which do not fully capture the physiological lipidated state or potential NTD-CTD coupling present in full-length APOE on discoidal lipoproteins. The absence of the C-terminal domain precludes formation of the antiparallel double-belt arrangement observed for lipidated APOE and may remodel surface exposure at helix 4. Where full-length coordinates are used (2L7B), we note that this model contains five C-terminal stabilizing substitutions and thus represents the engineered, non-lipidated construct. Accordingly, receptor–interface inferences here are state dependent and hypothesis generating; testing in lipidated, full-length contexts via construction of APOE3/APOE4 nanodiscs for the reassessment of helix 4 accessibility is an important direction for future work. A follow-up study focused on the stability of APOE2/APOE3 is considered as well.

## Figures and Tables

**Table 1:** Comparison of mutant ΔΔG values.

Mutation	APOE Variant	FoldX ΔΔG (kcal/mol)	DynaMut2 ΔΔG (kcal/mol)	DUET ΔΔG (kcal/mol)
G165W	APOE4	25.07 D	−0.82 D	−0.824 D
L155W	APOE4	17.93 D	−1.97 D	−2.273 D
L155W	APOE3	15.08 D	−2.11 D	−2.232 D
M108W	APOE4	16.22 D	−1.91 D	−1.595 D
M108W	APOE3	11.03 D	1.71 S	−1.603 D
D151I	APOE4	−4.42 S	1.04 S	0.742 S
D151F	APOE3	−3.81 S	−1.31 D	−0.905 D
D107M	APOE4	−4.49 S	1.13 S	0.598 S
D107L	APOE3	−4.25 S	0.32 S	0.73 S
C112R	APOE3	−0.53 S	−0.98 D	−0.912 D
R158C	APOE3	1.05 D	−1.23 D	−1.614 D
G165W	APOE3 (2L7B)	3.71 D	−1.3 D	−1.312 D
L155W	APOE3 (2L7B)	1.42 D	−2.01 D	−2.258 D
M108W	APOE3 (2L7B)	7.96 D	−1.82 D	−1.609 D
D151I	APOE3 (2L7B)	−0.32 S	0.2 S	0.758 S
D151F	APOE3 (2L7B)	6.31 D	−1.26 D	−1.144 D
D107M	APOE3 (2L7B)	3.08 D	−0.89 D	−0.938 D
D107L	APOE3 (2L7B)	−1.49 S	−1.13 D	−0.865 D

Notes: ΔΔG values represent predicted changes in protein stability upon mutation. For FoldX, positive values typically indicate destabilization (D), and negative values indicate stabilization (S). The ambiguity of the labels (D and S) between FoldX and Dynamut2/DUET arise from the definition of ΔΔG in opposite directions (ΔG_mut − ΔG_wt vs. ΔG_wt − ΔG_mut) or the normalization of outputs to a house convention. Dynamut2 and DUET use ΔΔG ≥ 0 = stabilizing and ΔΔG ≤ 0 = destabilizing, while FoldX reports ΔΔG > 0 = destabilizing and ΔΔG < 0 = stabilizing.

## Data Availability

The original contributions presented in this study are included in the article/Supplementary Materials. Further inquiries can be directed to the corresponding author(s).

## Supplementary Materials

The following supporting information can be downloaded at: https://www.mdpi.com/article/10.3390/genes16121509/s1, Table S1: ΔΔG values of all APOE3 (1NFN) mutations via FoldX; Table S2: ΔΔG values of all APOE4 (1GS9) mutations via FoldX; Table S3: ΔΔG values of all APOE3 (2L7B) mutations via FoldX; File S1: Custom Python script.

## Abbreviations

The following abbreviations are used in this manuscript:

AD	Alzheimer’s Disease
APOE	Apolipoprotein E
CTD	C-terminal Domain
DSSP	Dictionary of Secondary Structure Proteins
fs	Femtosecond
nm	Nanometer
NTD	N-terminal Domain
Rg	Radius of Gyration
RMSD	Root-Mean-Square Deviation
RMSF	Root-Mean-Square Fluctuation
SASA	Solvent-Accessible Surface Area
TREM2	Triggering Receptor Expressed on Myeloid Cells 2
WT	Wild Type
